# Intake of Vegetables and Fruits and the Risk of Cataract Incidence in a Japanese Population: The Japan Public Health Center-Based Prospective Study

**DOI:** 10.2188/jea.JE20190116

**Published:** 2021-01-05

**Authors:** Sayaka Adachi, Norie Sawada, Kenya Yuki, Miki Uchino, Motoki Iwasaki, Kazuo Tsubota, Shoichiro Tsugane

**Affiliations:** 1 Department of Ophthalmology, Keio University School of Medicine, Tokyo, Japan; 2 Epidemiology and Prevention Group, Center for Public Health Sciences, National Cancer Center, Tokyo, Japan

**Keywords:** food intake, cataract risk, food frequency questionnaire, prospective cohort study

## Abstract

**Background:**

Although the consumption of vegetables and fruits is reported to influence the risk of cataract, no prospective study of this association from Asia has yet appeared. Here, we investigated the association between vegetable and fruit intake and cataract incidence in a large-scale population-based prospective cohort study in Japan.

**Methods:**

This study included 32,387 men and 39,333 women aged 45–74 years who had no past history of cataract and had completed a dietary questionnaire of the Japan Public Health Center-based Prospective Cohort Study. The incidence of cataract was evaluated after 5-year follow-up. We used multiple logistic regression analyses to estimate the sex-specific odds ratios (ORs), with adjustment for confounding factors.

**Results:**

We identified 1,836 incident cataracts in 594 men and 1,242 women. In men, the OR for cataract was decreased with higher intake of vegetables (OR_Q5 vs Q1_, 0.77; 95% confidence interval [CI], 0.59–1.01; *P*_trend_ across quartile categories = 0.03) and cruciferous vegetables (OR_Q5 vs Q1_, 0.74; 95% CI, 0.57–0.96; *P*_trend_ = 0.02). In contrast, the OR for cataract was increased with higher intake of vegetables among women (OR_Q5 vs Q1_, 1.28; 95% CI, 1.06–1.53; *P*_trend_ = 0.01). Green and yellow vegetable and fruit intake were not associated with cataract in either sex.

**Conclusions:**

This study suggests that vegetables may reduce the risk of cataract in men, but not in women.

## INTRODUCTION

Age-related cataract is the leading cause of visual loss and blindness globally, with almost 20 million people affected.^1^ Cataract incidence increases with age,^2^ and since Japan leads the world in life expectancy,^3^ the incidence of cataract in Japan is also increasing. Visual impairment due to cataract increases the risk of falling^4^^,^^5^ and is associated with decreased cognitive function^6^^,^^7^ in elderly people. These findings warrant ongoing and focused epidemiological research on lifestyle prevention of cataract in the elderly.

Many studies have reported the association of cataract and foods, including vegetables.^8^^–^^15^ A meta-analysis indicated that higher consumption of vegetables might reduce cataract risk, mainly in American and European populations.^15^ Prospective cohort studies reported that the intake of vegetables and fruits, which include lutein, vitamin C, and vitamin E, may reduce the risk of cataracts,^9^^,^^10^^,^^13^ and that vegetarians had lower risk of cataract than meat eaters among British residents.^14^

Lens opacities in cataracts may occur as a result of lens protein damage by oxidative stress due to smoking,^16^ ultraviolet (UV) radiation exposure,^17^ steroid,^18^ diabetes mellitus,^19^^,^^20^ and high body mass index.^21^^–^^23^ Accordingly, it has been speculated that high doses of antioxidants, such as vitamin A, vitamin C, vitamin E, and β-carotene, may help prevent age-related cataract formation. Indeed, recent meta-analyses reported that such antioxidant intake might be associated with reduced cataract risk.^24^^–^^26^ Moreover, a recent study reported that cruciferous vegetables containing isothiocyanates protect lens cells against oxidative stress.^27^ Green and yellow vegetables are rich in carotenes and lutein, and a number of studies have reported an association between lutein and carotene and cataract risk.^8^^–^^10^ To date, however, no prospective study has reported the relationship between vegetables and fruits and cataract risk in Asia, although a previous case-control study in India reported that mean vegetable and fruit intakes were lower in cataract patients than in controls (*P* < 0.001).^28^

Here, we investigated the association between vegetable and fruit intake and incidence of cataract among middle-aged Japanese in a large-scale population-based prospective cohort study in Japan.

## MATERIALS AND METHODS

### Study cohort

The Japan Public Health Center-based Prospective (JPHC) Study was initiated in 1990 for cohort I and in 1993 for cohort II.^29^ Cohort I participants were residents aged 40–59 years in 1990 from five public health center areas (Iwate, Akita, Nagano, Okinawa-chubu, and Tokyo), while those of cohort II included residents aged 40–69 years in 1993 from six public health center areas (Ibaraki, Niigata, Kochi, Nagasaki, Okinawa-Miyako, and Osaka).

A questionnaire survey was carried out at baseline and at 5- and 10-year follow-up. The questionnaire was self-administered and included information on medical history and lifestyle, such as smoking and drinking habits and diet and vitamin supplement use at the time of the survey. We used the 5-year follow-up survey in place of the baseline survey as starting point for the present analysis because it provided more comprehensive information on dietary intake.

Ethics approval was obtained from the Institutional Review Board of the National Cancer Center and Keio University.

### Study population

Participant numbers were 103,880 in the 5-year (starting point) and 99,512 in the 10-year follow-up survey. Of these, 87,290 people participated in both surveys. We confirmed the diagnosis of age-related cataract using the following questions at the 5- and 10-year follow-up surveys: “Has a doctor ever told you that you had cataracts?”

We previously confirmed the validity of self-reported data on cataracts using a medical records review. We randomly selected 97 cases from 1,072 residents who self-reported a cataract diagnosis for cohort I. A total of 53 cases had permitted the review of medical records and had answers with regard to cataract diagnosis for validation study. We compared the self-reported response ‘I have been diagnosed as having cataracts’ with medical records and found that the positive predictive value of a self-reported diagnosis of age-related cataract was confirmed in 49 of 53 self-reporters (92.5%).^30^

We excluded 4,361 participants who had been diagnosed with cataract at the starting point. Of the remainder, 2,321 were diagnosed with age-related cataract at the 10-year follow-up survey (ie, 5 years later) and confirmed to have age-related cataract. Of 82,929 participants, we excluded 1,308 who had missing information on the frequency of vegetable intake; 4,078 in either the upper or lower 2.5% of sex-specific total energy intake; and 5,823 with a history of cancer, cardiovascular diseases, and diabetes mellitus, leaving 71,720 for final analysis, consisting of 32,387 men and 39,333 women.

### Food frequency questionnaire (FFQ)

The 5-year follow-up survey used food frequency questionnaires that were designed to estimate dietary habits. They included questions on the intake of 138 food items,^31^^,^^32^ including total fruits (16 fruit items), total vegetables (30 vegetable items), cruciferous vegetables (11 vegetable items), and green and yellow vegetables (16 vegetable items) during the previous year.

The FFQ inquired about the usual consumption of 30 vegetables (carrots, spinach, pumpkins, cabbage, Chinese cabbage, Chinese radishes, pickled Chinese radishes, pickled green leafy vegetables, pickled plums, pickled Chinese cabbage, pickled cucumbers, pickled eggplant, sweet pepper, tomatoes, Chinese chives, garland chrysanthemums, komatsuna, broccoli, onions, cucumbers, bean sprouts, snap beans, lettuce, chingensai, leaf mustard, bitter gourd, [Swiss] chard, loofah, mugwort, and tomato juice). Total cruciferous vegetables consisted of eight cruciferous vegetables and three pickled cruciferous vegetables: cabbage, Chinese radish, broccoli, komatsuna, Chinese leaves, pak choi, leaf mustard, and Swiss chard as cruciferous vegetables adopted in the IARC Handbooks of Cancer Prevention,^33^ and pickled Chinese radish, rape and leaf mustard, and Chinese leaves as pickled cruciferous vegetables. Green and yellow vegetables consisted of 10 green and yellow vegetables and one pickled green and yellow vegetable: broccoli, carrots, Chinese chives, chingensai, crown daisy, green beans, komatsuna, leaf mustard, mugwort, pickled green leafy vegetables, pumpkin, spinach, sweet pepper, Swiss chard, tomato, and tomato juice. Total fruits constituted of 16 fruits: papaya, mandarin oranges, other oranges, apples, persimmons, strawberries, grapes, melons, watermelon, peaches, pears, kiwifruit, pineapple, bananas, 100% orange juice, and 100% apple juice.

Each food item was given nine options of consumption frequency, ranging from rarely (less than once a month) to seven or more times a day. Standard portion sizes for each food item were divided into the three options of small (less than half the standard serving size), medium (standard serving size), or large (more than one and half times the standard size). The daily intake of each food was calculated by multiplying the daily consumption frequency and relative portion size for each food item.

The intake of each food item was adjusted for total energy intake using the residual method.^34^ Food intakes were divided into quartiles. For the validity of food intake measurements from the FFQ and 28-day dietary records, Spearman’s rank correlation coefficients were 0.22 in men and 0.32 in women for vegetable intake and 0.41 in men and 0.23 in women for fruit intake in cohort I^31^; and 0.35 in men and 0.43 in women for vegetable intake and 0.50 in men and 0.30 in women for fruit intake in cohort II.^32^ These results reveal that the FFQ can be used to rank individuals according to food intake in the JPHC Study.

### Statistical analysis

Multiple logistic-regression analyses were used to calculate the sex-specific odds ratios (ORs) and 95% confidence intervals (CIs) of cataract incidence according to quartiles of total vegetables, cruciferous vegetables, green and yellow vegetables, and fruits.

Multivariate adjustment used covariates with risk factors known or suspected to affect cataract incidence.^16^^,^^23^^,^^35^ These were adjusted for age, area, and the following potential confounding factors: body-mass index (BMI), calculated as weight (kg)/height squared (m^2^) and grouped into four categories (<21.0, 21.0–22.9, 23.0–24.9, and ≥25.0 kg/m^2^); smoking status (non-smokers who did not have a history of smoking, and smokers who smoked currently or had smoked in the past); weekly alcohol intake (g/week), using four levels of consumption in men (non- and occasional drinkers, and drinkers of 1–149 g/week, 150–299 g/week, 300–499 g/week, and ≥450 g/week) and in women (non- and occasional drinkers, and drinkers of ≥1 g/week); total fruit intake, which was an adjustment factor in the analysis of total vegetable, cruciferous vegetable, and green and yellow vegetable intake (otherwise, total vegetable intake was used as an adjustment factor in the analysis of total fruit intake); vitamin supplement intake (yes or no); and fundus photographic examination (yes or no). Furthermore, we calculated *P* interaction values using a likelihood-ratio test to compare logistic models with and without cross-product terms for smoking (non-smokers or smokers) and age (<60 or ≥60 years).

All statistical analyses were performed with SAS version 9.4 (SAS Institute, Cary, NC, USA).

## RESULTS

Table 1 shows the baseline characteristics of participants according to quartile of energy-adjusted total vegetable intake. Intake of vegetables, cruciferous vegetables, green and yellow vegetables, and fruits tended to be higher in older people; in those with higher BMI, those who never or only occasionally drank alcohol, and non-smokers; and in those who underwent fundus photographic examinations.

**Table 1.** Baseline characteristics of participants according to quartile of vegetable intake by sex

Men	Vegetable intake by quartile				
	
	Quartile 1	Quartile 2	Quartile 3	Quartile 4	*P value*
	0–110.7	110.7–166.4	166.4–242.7	>241.7	
Participants	8,096	8,097	8,097	8,097	
Age, years, mean (SD)	49.7 (7.4)	50.3 (7.4)	51.3 (7.6)	52.5 (7.6)	<0.0001
BMI, kg/m^2^, mean (SD)	23.4 (2.9)	23.5 (2.7)	23.6 (2.8)	23.7 (2.9)	<0.0001
Total energy intake, kcal/day, mean (SD)	2,166.9 (647.4)	2,235.1 (629.1)	2,214.9 (620.0)	2,157.8 (630.4)	<0.0001
Vegetable intake, g/day, mean (SD)	78.5 (39.4)	150.2 (52.6)	215.9 (74.6)	367.1 (190.9)	<0.0001
Cruciferous vegetable intake, g/day, mean (SD)	33.4 (21.0)	61.4 (30.8)	86.3 (42.2)	145.2 (99.0)	<0.0001
Green and yellow vegetable intake, g/day, mean (SD)	30.8 (17.3)	60.8 (22.9)	90.2 (30.7)	161.5 (86.0)	<0.0001
Fruit intake, g/day, mean (SD)	121.3 (143.4)	167.3 (147.8)	207.5 (169.5)	245.7 (207.6)	<0.0001
Smoking status, %					<0.0001
Non-smokers	30.7	33.6	37.2	41.9	
Smokers	69.3	66.4	62.8	58.1	
Alcohol consumption, %					<0.0001
Non and occasional drinkers	20.1	21.1	24.6	29.9	
1–149 g/week	19.7	23.8	26.8	29.5	
150–299 g/week	17.6	19.6	20.5	19.8	
300–449 g/week	17.7	17	14.9	12.1	
≥450 g/week	25	18.5	13.2	8.7	
Supplement intake, %	9	9.8	10.4	11.2	<0.0001
Fundus photographic examination, %	39.5	43.1	45.3	46.4	<0.0001

Women	Vegetable intake by quartile				
	
	Quartile 1	Quartile 2	Quartile 3	Quartile 4	*P value*
	0–139.9	139.9–199.7	199.7–278.8	>278.8	

Participants	9,833	9,833	9,834	9,833	
Age, years, mean (SD)	50.4 (7.7)	50.7 (7.6)	51.3 (7.5)	52.2 (7.5)	<0.0001
BMI, kg/m^2^, mean (SD)	23.4 (3.2)	23.4 (3.1)	23.5 (3.1)	23.6 (3.1)	<0.0001
Total energy intake, kcal/day, mean (SD)	1,882.2 (603.1)	1,902.7 (554.3)	1,881.8 (531.2)	1,851.7 (542.4)	<0.0001
Vegetable intake, g/day, mean (SD)	107.9 (53.1)	183.7 (67.7)	251.5 (89.1)	411.4 (209.5)	<0.0001
Cruciferous vegetable intake, g/day, mean (SD)	44.3 (27.0)	74.6 (37.0)	100.7 (47.9)	164.7 (109.3)	<0.0001
Green and yellow vegetable intake, g/day, mean (SD)	42.9 (19.7)	74.4 (24.7)	105.7 (33.0)	178.4 (86.3)	<0.0001
Fruit intake, g/day, mean (SD)	203.9 (193.9)	249.1 (199.1)	272.4 (202.6)	303.9 (233.9)	<0.0001
Smoking status, %					<0.0001
Non-smokers	92.3	93.5	94.4	95.5	
Smokers	7.7	6.5	5.6	4.5	
Alcohol consumption, %					<0.0001
Non and occasional drinkers	78.2	78.7	80.7	84.0	
≥1 g/week	21.8	21.3	19.4	16.0	
Supplement intake, %	13.7	15.2	15.2	15.0	0.007
Fundus photographic examination, %	39.7	45.4	47.9	50.2	<0.0001

BMI, body mass index; SD, standard deviation.

Table 2 shows the age- and area-adjusted ORs and multivariate ORs with 95% CIs for cataract incidence according to energy-adjusted total vegetable, cruciferous vegetable, green and yellow vegetable, and fruit intake by quartile. In men, the multivariate ORs for the highest versus lowest quartile of total vegetable and cruciferous vegetable intake were 0.77 (95% CI, 0.59–1.01) (*P* for trend = 0.03) and 0.74 (95% CI, 0.57–0.96) (*P* for trend = 0.02), respectively. The multivariate ORs for the highest versus lowest quartile of green and yellow vegetable and total fruit intake were 0.85 (95% CI, 0.66–1.11) and 1.13 (95% CI, 0.85–1.49), respectively. In women, in contrast, the results for total vegetable and cruciferous vegetable intakes showed the opposite results, with a multivariate OR for the highest quartile of energy-adjusted total vegetable intake compared with the lowest of 1.28 (95% CI, 1.06–1.53) (*P* for trend = 0.01). Further, the multivariate OR of cruciferous vegetables was positively associated with cataract risk, albeit with no significant difference in trend analysis. A multivariate OR for the highest quartile of green and yellow vegetable intake was positively associated with cataract, but without statistical significance. Total fruit intake also tended to be positively associated, at 1.15 (95% CI, 0.96–1.39), but without statistical significance. Furthermore, we adjusted for vitamin C and vitamin E intake in the same model, but the results did not change. Additionally, we further adjusted for energy intake in the same model, and the results were not substantially changed.

**Table 2.** Age- and area-adjusted odds ratios and multivariate odds ratios for cataract incidence according to total vegetable, cruciferous vegetable, green and yellow vegetable and fruit intake category in men and women

	Men						

		Quartile category				*P for trend*	Per 50 g/day increment

		Quartile 1	Quartile 2	Quartile 3	Quartile 4		
		OR (95% CI)	OR (95% CI)	OR (95% CI)	OR (95% CI)		OR (95% CI)
Total vegetable intake							
	Range, g	0–110.66	110.7–166.4	166.4–242.7	>241.7		
	Number of participants	8,096	8,097	8,097	8,097		
	Number of cases	128	146	151	169		
	Model 1	1.00	1.07 (0.84–1.36)	0.98 (0.77–1.25)	0.97 (0.77–1.24)	0.64	
	Model 2	1.00	0.98 (0.76–1.27)	0.85 (0.65–1.10)	0.77 (0.59–1.01)	0.03	0.95 (0.91–0.99)
Cruciferous vegetable intake							
	Range, g	0–39.3	39.3–63.7	63.7–96.8	>96.8		
	Number of participants	8,096	8,097	8,097	8,097		
	Number of cases	139	136	152	167		
	Model 1	1.00	0.92 (0.72–1.17)	0.93 (0.73–1.18)	0.87 (0.68–1.10)	0.27	
	Model 2	1.00	0.89 (0.69–1.14)	0.83 (0.64–1.07)	0.74 (0.57–0.96)	0.02	0.89 (0.82–0.97)
Green and yellow vegetable intake							
	Range, g	0–40.9	40.9–71.2	71.2–111.6	>11.6		
	Number of participants	8,096	8,097	8,097	8,097		
	Number of cases	137	142	147	168		
	Model 1	1.00	1.01 (0.79–1.28)	0.99 (0.78–1.26)	1.02 (0.81–1.29)	0.89	
	Model 2	1.00	0.95 (0.74–1.22)	0.90 (0.70–1.17)	0.85 (0.66–1.11)	0.21	0.95 (0.89–1.02)
Total fruit intake							
	Range, g	0–72.9	72.9–140.4	140.4–230.3	>230.3		
	Number of participants	8,096	8,097	8,097	8,097		
	Number of cases	126	126	172	170		
	Model 1	1.00	0.95 (0.74–1.22)	1.21 (0.95–1.53)	1.08 (0.85–1.37)	0.24	
	Model 3	1.00	0.93 (0.71–1.22)	1.22 (0.94–1.59)	1.13 (0.85–1.49)	0.17	1.02 (0.99–1.05)

	Women						
	
		Quartile category				*P for trend*	Per 50 g/day increment
	
		Quartile 1	Quartile 2	Quartile 3	Quartile 4		
		OR (95% CI)	OR (95% CI)	OR (95% CI)	OR (95% CI)		OR (95% CI)

Total vegetable intake							
	Range, g	0–139.9	139.9–199.7	199.7–278.8	>278.8		
	Number of participants	9,833	9,833	9,834	9,833		
	Number of cases	246	292	327	377		
	Model 1	1.00	1.19 (1.00–1.41)	1.30 (1.10–1.54)	1.43 (1.21–1.69)	<0.0001	
	Model 2	1.00	1.13 (0.94–1.36)	1.15 (0.95–1.38)	1.28 (1.06–1.53)	0.01	1.02 (1.00–1.04)
Cruciferous vegetable intake							
	Range, g	0–50.0	50.0–76.6	76.6–113.3	≧113.3		
	Number of participants	9,833	9,833	9,834	9,833		
	Number of cases	244	304	327	367		
	Model 1	1.00	1.26 (1.06–1.49)	1.27 (1.07–1.50)	1.33 (1.12–1.58)	0.002	
	Model 2	1.00	1.21 (1.01–1.46)	1.19 (0.98–1.44)	1.23 (1.02–1.48)	0.06	1.04 (0.99–1.09)
Green and yellow vegetable intake							
	Range, g	0–54.0	54.0–85.2	85.2–127.9	>127.9		
	Number of participants	9,833	9,833	9,834	9,833		
	Number of cases	274	281	327	360		
	Model 1	1.00	1.08 (0.91–1.28)	1.21 (1.03–1.43)	1.28 (1.09–1.51)	0.001	
	Model 2	1.00	0.99 (0.82–1.18)	1.09 (0.91–1.30)	1.08 (0.90–1.29)	0.25	1.00 (0.96–1.05)
Total fruit intake							
	Range, g	0–125.6	125.6–203.8	203.8–306.8	>306.8		
	Number of participants	9,833	9,833	9,834	9,833		
	Number of cases	270	279	344	349		
	Model 1	1.00	1.01 (0.85–1.20)	1.22 (1.03–1.44)	1.20 (1.02–1.43)	0.007	
	Model 3	1.00	0.98 (0.82–1.18)	1.13 (0.94–1.36)	1.15 (0.96–1.39)	0.06	1.01 (0.99–1.03)

CI, confidence interval; OR, odds ratio.

Model 1 was adjusted for age and area.

Model 2 was adjusted for age, area, smoking status, alcohol consumption, body mass index, fruit intake, supplement intake, and fundus photographic examination.

Model 3 was adjusted for age, area, smoking status, alcohol consumption, body mass index, vegetable intake, supplement intake, and fundus photographic examination.

Table 3 shows the multivariate ORs with 95% CIs by smoking status for cataract incidence according to energy-adjusted total vegetable, cruciferous vegetable, green and yellow vegetable, and fruit intake by quartile. The multivariate OR for the highest quartile of total vegetable intake compared with the lowest was further reduced in smoking compared with total men (OR 0.67; 95% CI, 0.48–0.96). For cruciferous vegetables in men, the multivariate OR for the highest quartile of cruciferous vegetable intake compared with the lowest was further reduced in smoking compared with total men (OR 0.71; 95% CI, 0.50–1.00). For intake of green and yellow vegetables in smoking men, the multivariate OR tended to be negatively associated (OR 0.74; 95% CI, 0.53–1.05), but without statistical significance. For total fruits, the multivariate OR for the highest quartile compared with the lowest was 1.32 (95% CI, 0.92–1.90) in smoking men. In contrast, the results for total vegetables in women showed the opposite results, with a multivariate OR for the highest quartile of total vegetable intake compared with the lowest quartile of 1.27 (95% CI, 1.06–1.54) in non-smoking women. For cruciferous vegetables in women, the OR in non-smoking women was positively associated with higher intake of cruciferous vegetables, with a cataract incidence of 1.23 (95% CI, 1.01–1.48), but this was not statistically significant in trend analysis. For intakes of green and yellow vegetables and fruits in women, the ORs for the highest quartile compared with the lowest were 1.08 (95% CI, 0.90–1.31) and 1.15 (95% CI, 0.95–1.39), respectively, in non-smoking women. The interaction *P* values were not statistically significant.

**Table 3.** Multivariate odds ratios for cataract incidence according to total vegetable, cruciferous vegetable, green and yellow vegetable, and fruit intake category in men and women by smoking status

	Men						

		Quartile category				*P for trend*	*Interaction P-value*

		Quartile 1	Quartile 2	Quartile 3	Quartile 4		
		OR (95% CI)	OR (95% CI)	OR (95% CI)	OR (95% CI)		
Total vegetable intake							0.31
Non-smokers	Number of participants	2,360	2,597	2,875	3,212		
	Number of cases	41	53	67	73		
	Model 1	1.00	1.03 (0.68–1.58)	1.06 (0.70–1.60)	0.95 (0.62–1.45)	0.78	
Smokers	Number of participants	5,330	5,139	4,857	4,462		
	Number of cases	84	87	75	80		
	Model 1	1.00	0.96 (0.70–1.32)	0.73 (0.52–1.03)	0.67 (0.48–0.96)	0.01	
Cruciferous vegetable intake							0.67
Non-smokers	Number of participants	2,581	2,706	2,829	2,928		
	Number of cases	53	56	61	64		
	Model 1	1.00	0.84 (0.57–1.24)	0.84 (0.57–1.25)	0.77 (0.52–1.14)	0.23	
Smokers	Number of participants	5,110	5,039	4,899	4,740		
	Number of cases	80	77	83	86		
	Model 1	1.00	0.92 (0.66–1.27)	0.81 (0.58–1.13)	0.71 (0.50–1.00)	0.04	
Green and yellow vegetable intake							0.16
Non-smokers	Number of participants	2,337	2,588	2,830	3,289		
	Number of cases	41	50	66	77		
	Model 1	1.00	1.03 (0.67–1.58)	1.18 (0.78–1.78)	1.07 (0.70–1.63)	0.67	
Smokers	Number of participants	5,311	5,148	4,886	4,443		
	Number of cases	91	82	73	80		
	Model 1	1.00	0.92 (0.67–1.27)	0.77 (0.55–1.07)	0.74 (0.53–1.05)	0.06	
Total fruit intake							0.88
Non-smokers	Number of participants	2,150	2,608	2,973	3,313		
	Number of cases	42	43	77	72		
	Model 2	1.00	0.84 (0.54–1.30)	1.17 (0.77–1.76)	0.94 (0.60–1.46)	0.84	
Smokers	Number of participants	5,559	5,142	4,749	4,338		
	Number of cases	74	77	86	89		
	Model 2	1.00	1.03 (0.73–1.45)	1.29 (0.92–1.81)	1.32 (0.92–1.90)	0.07	

	Women						

		Quartile category				*P for trend*	*Interaction P-value*

		Quartile 1	Quartile 2	Quartile 3	Quartile 4		
		OR (95% CI)	OR (95% CI)	OR (95% CI)	OR (95% CI)		

Total vegetable intake							0.21
Non-smokers	Number of participants	8,473	8,685	8,750	8,858		
	Number of cases	212	248	281	338		
	Model 1	1.00	1.08 (0.89–1.30)	1.14 (0.95–1.38)	1.27 (1.06–1.54)	0.008	
Smokers	Number of participants	704	607	519	414		
	Number of cases	12	24	13	11		
	Model 1	1.00	2.17 (1.05–4.49)	1.12 (0.49–2.59)	1.21 (0.51–2.88)	0.89	
Cruciferous vegetable intake							0.56
Non-smokers	Number of participants	8,520	8,736	8,743	8,767		
	Number of cases	208	265	283	323		
	Model 1	1.00	1.20 (0.99–1.45)	1.17 (0.97–1.41)	1.23 (1.01–1.48)	0.07	
Smokers	Number of participants	711	553	551	429		
	Number of cases	13	15	20	12		
	Model 1	1.00	1.29 (0.59–2.79)	1.77 (0.84–3.72)	1.19 (0.51–2.75)	0.46	
Green and yellow vegetable intake							0.86
Non-smokers	Number of participants	8,374	8,731	8,790	8,871		
	Number of cases	234	241	284	320		
	Model 1	1.00	0.96 (0.80–1.16)	1.07 (0.89–1.28)	1.08 (0.90–1.31)	0.24	
Smokers	Number of participants	688	563	508	485		
	Number of cases	14	17	16	13		
	Model 1	1.00	1.49 (0.71–3.16)	1.39 (0.64–3.00)	1.00 (0.44–2.25)	0.95	
Total fruit intake							0.93
Non-smokers	Number of participants	8,375	8,754	8,813	8,824		
	Number of cases	223	247	298	311		
	Model 2	1.00	1.00 (0.82–1.21)	1.14 (0.94–1.38)	1.15 (0.95–1.39)	0.07	
Smokers	Number of participants	888	556	429	371		
	Number of cases	23	12	12	13		
	Model 2	1.00	0.75 (0.36–1.57)	0.92 (0.43–1.98)	1.26 (0.59–2.70)	0.59	

CI, confidence interval; OR, odds ratio.

Model 1 was adjusted for age, area, alcohol consumption, body mass index, fruit intake, supplement intake, and fundus photographic examination.

Model 2 was adjusted for age, area, alcohol consumption, body mass index, vegetable intake, supplement intake, and fundus photographic examination.

Table 4 shows the multivariate ORs with 95% CIs stratified by age for cataract incidence according to total vegetable, cruciferous vegetable, green and yellow vegetable, and fruit intake by quartile. The multivariate ORs of total vegetable and cruciferous vegetable intake compared with the lowest was further reduced for men in the ≥60 years age group than for those in the <60 years age group (OR_Q5 vs Q1_, 0.66; 95% CI, 0.48–0.92) for total vegetable, *P* for interaction = 0.04 and OR_Q5 vs Q1_, 0.63; 95% CI, 0.46–0.85) for cruciferous vegetable, *P* for interaction = 0.007). Meanwhile, no significant differences by age were found for green and yellow vegetable and total fruit intake in men (*P* for interaction = 0.71 for green and yellow vegetable intake and *P* for interaction = 0.86 for total fruit intake). Moreover, significant differences by age among women were also not found.

**Table 4.** Multivariate odds ratios for cataract incidence according to total vegetable, cruciferous vegetable, green and yellow vegetable, and fruit intake category in men and women stratified by age

	Men						

		Quartile category				*P for trend*	Interaction *P*-value

		Quartile 1	Quartile 2	Quartile 3	Quartile 4		
		OR (95% CI)	OR (95% CI)	OR (95% CI)	OR (95% CI)		
Total vegetable intake							0.03
Age <60 years	Number of participants	5,917	5,663	5,251	4,652		
	Number of cases	42	44	45	50		
	Model 1	1.00	1.08 (0.70–1.68)	1.11 (0.71–1.75)	1.23 (0.77–1.95)	0.39	
Age ≥60 years	Number of participants	2,179	2,434	2,846	3,445		
	Number of cases	86	102	106	119		
	Model 1	1.00	0.96 (0.71–1.31)	0.79 (0.57–1.08)	0.66 (0.48–0.92)	0.005	
Cruciferous vegetable intake							0.007
Age <60 years	Number of participants	6,002	5,727	5,263	4,491		
	Number of cases	43	43	52	43		
	Model 1	1.00	1.11 (0.72–1.71)	1.34 (0.87–2.06)	1.36 (0.86–2.16)	0.13	
Age ≥60 years	Number of participants	2,094	2,370	2,834	3,606		
	Number of cases	96	93	100	124		
	Model 1	1.00	0.81 (0.59–1.10)	0.68 (0.50–0.93)	0.63 (0.46–0.85)	0.002	
Green and yellow vegetable intake							0.71
Age <60 years	Number of participants	5,704	5,559	5,346	4,874		
	Number of cases	50	42	37	52		
	Model 1	1.00	0.78 (0.51–1.20)	0.67 (0.43–1.06)	0.89 (0.57–1.39)	0.53	
Age ≥60 years	Number of participants	2,392	2,538	2,751	3,223		
	Number of cases	87	100	110	80		
	Model 1	1.00	0.92 (0.67–1.27)	0.77 (0.55–1.07)	0.74 (0.53–1.05)	0.06	
Total fruit intake							0.86
Age <60 years	Number of participants	5,828	5,575	5,293	4,787		
	Number of cases	52	37	45	47		
	Model 2	1.00	0.74 (0.48–1.16)	0.98 (0.63–1.51)	1.14 (0.72–1.79)	0.41	
Age ≥60 years	Number of participants	2,268	2,522	2,804	3,310		
	Number of cases	74	89	127	123		
	Model 2	1.00	1.08 (0.77–1.53)	1.41 (1.01–.97)	1.18 (0.83–1.68)	0.41	

	Women						

		Quartile category				*P for trend*	Interaction *P*-value

		Quartile 1	Quartile 2	Quartile 3	Quartile 4		
		OR (95% CI)	OR (95% CI)	OR (95% CI)	OR (95% CI)		

Total vegetable intake							0.40
Age <60 years	Number of participants	6,848	6,743	6,472	6,029		
	Number of cases	86	115	113	127		
	Model 1	1.00	1.30 (0.97–1.73)	1.23 (0.91–1.65)	1.43 (1.06–1.93)	0.04	
Age ≥60 years	Number of participants	2,985	3,090	3,362	3,804		
	Number of cases	160	177	214	250		
	Model 1	1.00	1.04 (0.82–1.32)	1.12 (0.89–1.42)	1.26 (1.00–1.58)	0.04	
Cruciferous vegetable intake							0.25
Age <60 years	Number of participants	6,979	6,854	6,424	5,835		
	Number of cases	93	117	121	110		
	Model 1	1.00	1.30 (0.97–1.72)	1.36 (1.02–1.82)	1.32 (0.97–1.79)	0.08	
Age ≥60 years	Number of participants	2,854	2,979	3,410	3,998		
	Number of cases	151	187	206	257		
	Model 1	1.00	1.16 (0.92–1.48)	1.14 (0.90–1.45)	1.26 (1.00–1.59)	0.08	
Green and yellow vegetable intake							0.053
Age <60 years	Number of participants	6,608	6,733	6,529	6,222		
	Number of cases	89	101	128	123		
	Model 1	1.00	1.04 (0.78–1.40)	1.33 (1.00–1.76)	1.21 (0.90–1.63)	0.09	
Age ≥60 years	Number of participants	3,225	3,100	3,305	3,611		
	Number of cases	185	180	199	237		
	Model 1	1.00	0.95 (0.75–1.19)	0.97 (0.77–1.21)	1.06 (0.85–1.33)	0.53	
Total fruit intake							0.08
Age <60 years	Number of participants	6,700	6,666	6,433	6,293		
	Number of cases	98	92	114	137		
	Model 2	1.00	0.89 (0.66–1.20)	1.13 (0.84–1.51)	1.38 (1.03–1.84)	0.008	
Age ≥60 years	Number of participants	3,133	3,167	3,401	3,540		
	Number of cases	172	187	230	212		
	Model 2	1.00	1.06 (0.84–1.34)	1.15 (0.91–1.45)	1.04 (0.82–1.33)	0.68	

CI, confidence interval; OR, odds ratio.

Model 1 was adjusted for age, area, smoking status, alcohol consumption, body mass index, fruit intake, supplement intake, and fundus photographic examination.

Model 2 was adjusted for age, area, smoking status, alcohol consumption, body mass index, vegetable intake, supplement intake, and fundus photographic examination.

These results for men and women did not change following stratification by vitamin supplement intake (data not shown).

## DISCUSSION

In this prospective cohort study of the relationship between vegetable and fruit intake and incidence of cataract, we found an inverse association between higher intake of total vegetables and cruciferous vegetables and cataract incidence in men, but not in women. Our findings also revealed that there was no association between green and yellow vegetable and fruit intake and cataract risk for Japanese. To our knowledge, this is the first study in an Asian population to report the association of vegetable, cruciferous vegetable, green and yellow vegetable, and fruit intake with cataract risk.

A meta-analysis of the association between vegetable consumption and risk of age-related cataract that included nine articles involving 6,464 cataract cases and 112,447 participants found that higher vegetable consumption decreased the risk of cataract.^15^ In stratified analysis by study design, cohort studies from America and Europe showed inverse associations between vegetable consumption and cataract risk (summary RR 0.871; 95% CI, 0.791–0.959). Previous studies reported that intake of broccoli and spinach, included in cruciferous vegetables, reduced cataract risk,^8^^–^^10^ including a case-control study in Italy that found higher intake of cruciferous vegetables associated with decreased risk of cataract (summary OR 0.5; 95% CI, 0.3–0.8).^8^ Our finding of an inverse association between vegetable and cruciferous vegetable intake and cataract in men is consistent with these previous findings. Vegetables contain antioxidants, including vitamins, β carotene, phytoestrogens, folate and dietary fiber, which may prevent cataract progression.^36^^,^^37^ These components are involved in biological processes that may alter the structure of cataracts, such as DNA methylation, oxidative stress, and protection against DNA damage.^38^

Previous studies showed an inverse or no association between carotene and lutein, which are abundant in green and yellow vegetables, and the risk of cataract,^9^^,^^10^^,^^14^^,^^39^^,^^40^ and showed that green and yellow vegetable intake reduced cataract risk.^8^^–^^10^ In this study, there was no significant difference between higher intake of green and yellow vegetables and the incidence of cataract. Brown et al reported that a high frequency of carrot and tomato intake was not related to cataract risk,^9^ whereas Tavani et al reported that high intake of spinach and tomatoes decreased cataract risk.^8^ The Blue Mountains Eye Study showed that there was no association between intake of green and yellow vegetables and cataract risk.^11^ In other words, not all green and yellow vegetables appear to reduce the risk of cataract.

It has been reported that fruit intake might be associated with a decreased risk of cataract incidence.^39^^–^^41^ Theodoropoulou et al^39^ reported a significant inverse association between monthly frequency of fruit consumption and risk of cataract in a case-control study. Pastor-Valero et al^40^ and Christen et al^41^ reported that increasing quartiles of combined fruit and vegetable intake were related with a reduction in cataract risk. While these previous analyses of higher intake of all fruits or all vegetables separately were similarly shown to decrease the risk of cataract, albeit without significant differences, our present findings showed no obvious relation between total fruit intake and cataract incidence risk among subjects of either sex in our study, despite the relatively high antioxidant content of fruits.^36^^,^^37^ According to Food and Agriculture Organization of the United Nations data, fruit consumption in Japan (average 144.8 g/capita/day) is the lowest in developed countries, and one-third or less than that in the Netherlands (average 482.5 g/capita/day), the country with the highest fruit consumption.^42^ Since average Japanese fruit intake is low, there may have been no association between fruit intake and cataract incidence, although the average intake of fruits in our highest category was over 200 g.

Because previous reports showed that smoking has effects on cataracts and is a cause of oxidative stress,^43^^–^^47^ we stratified the results by smoking status. Results showed a clearer association between total cruciferous vegetable intake and cataract incidence among smoking men. Cruciferous vegetables, such as broccoli, are rich in carotenoids, vitamin C, vitamin E, vitamin K, folate, and minerals. A previous study showed that cruciferous vegetables contain isothiocyanates, such as sulforaphane, which protect lens cells against oxidative stress.^27^ Cruciferous vegetables have antioxidant activity and may act to antagonize the oxidative action of smoking, and possibly to inhibit cataract incidence.

However, the percentage of cataract incidence was lower in smokers (1.6% in men and 2.7% in women) than non-smokers (2.1% in men and 3.1% in women) in both sexes, as shown in Table 3. Smokers might be less likely to consult an ophthalmologist than non-smokers due to a lack of health consciousness. Therefore, we cannot rule out the possibility that the results in smokers were overestimated due to detection bias, and the results in non-smokers may be more certain.

Contrary to our expectations, we found a positive association between total vegetable and cruciferous vegetable intake and cataract in women. Given the higher incidence of cataract in women than men in this study, this might be partly explained by the difference between sexes in the percentage of participants receiving medical advice. There are few subjective symptoms in the early development of cataracts, and many cataracts progress and develop symptoms slowly. Further, cataracts are often not diagnosed without a doctor visit. The Japanese Ministry of Health, Labour and Welfare reported in a patient survey of eligible persons using medical facilities throughout the country that women are more likely to consult a doctor than men.^48^ This result is consistent with the report by Simon et al.^49^ Accordingly, if participants visit a doctor, early lens opacities are also likely to be diagnosed. Since we had no information from consultations with doctors, we used information from fundus photographic examination at health check-ups as surrogate information. Although we adjusted data and stratified subjects by the presence of fundus photographic examination, the positive association in women was not changed. Correspondingly, the positive association between total vegetable and cruciferous vegetable intake and cataract risk for men in the <60 years age group is similar to the results in women. Like women, relatively younger men with a higher intake of vegetables might be more likely to consult a doctor at a health check-up or comprehensive medical examination due to increased health consciousness. Therefore, the results in women and men aged <60 years might be overestimated due to detection bias. Since we could not adjust by the number of persons who actually consulted doctors, we cannot remove this detection bias. A second consideration is that women generally have a higher incidence of cataract than men in the same age group due to the decrease in estrogen which follows menopause, which causes cataract progression.^50^^,^^51^ In other words, the lens is protected in women by the antioxidant action of estrogen until menopause. In our study, higher consumption of vegetables and cruciferous vegetables was associated with older age. We were, therefore, unable to eliminate the potential biases and confounding factors arising from this difference. Further study of the cause of these sex differences in the influence of vegetable and cruciferous vegetable intake and cataract incidence is anticipated.

We agree that some randomized trials of antioxidant vitamin supplements have not shown protective effects against cataract, and further studies of antioxidant vitamins supplements have not been recommended.^52^ However, this study might be meaningful in revealing the relationship between vegetable and fruit intake and cataract risk in daily life.

The strengths of the present study include its large number of participants of both sexes; population-based prospective cohort design; limited localized bias, with inclusion of 11 public health center areas nationwide; use of a validated FFQ; and adjustment or stratification for potentially important confounding factors. However, the study also has several limitations. First, we had no source of information for cataract diagnosis other than the questionnaire, and might therefore have underestimated cataract incidence due to false-negative cases who had not visited an ophthalmology clinic and were not diagnosed with cataract. These sources of detection bias may have led to an underestimation of the overall incidence of age-related cataract. At the same time, this detection bias may have affected our results as mentioned above. In previous studies in which information on cataract was obtained using medical records or evaluation via ophthalmic examination, and not from self-report data, some reports showed an inverse association in women.^40^^,^^41^ We might not be able to remove this detection bias. Second, evaluation of dietary intake occurred at only one time point: the second survey, which was the baseline of this study. Repeated assessment of long-term dietary intake before disease onset is more likely to predict exposure conditions. Third, the follow-up period of 5 years was relatively short for a disease with such a protracted natural history. Fourth, we analyzed the data after excluding subjects with a history of diabetes mellitus; however, we did not exclude cases having diabetic mellitus during follow-up, and did not adjust for glucose/HbA1c level because data on these variables was not available. Finally, we were unable to remove the possibility of unmeasured and residual confounding factors.

In conclusion, the results of this prospective large-cohort study suggested that the intake of vegetables and cruciferous vegetables may reduce the risk of cataract incidence among Japanese men.
